# Cortical Dysplasia and the mTOR Pathway: How the Study of Human Brain Tissue Has Led to Insights into Epileptogenesis

**DOI:** 10.3390/ijms23031344

**Published:** 2022-01-25

**Authors:** Wei Shern Lee, Sara Baldassari, Sarah E. M. Stephenson, Paul J. Lockhart, Stéphanie Baulac, Richard J. Leventer

**Affiliations:** 1 Bruce Lefroy Centre, Murdoch Children’s Research Institute, Parkville 3052, Australia; weishern.lee@mcri.edu.au (W.S.L.); sarah.stephenson@mcri.edu.au (S.E.M.S.); paul.lockhart@mcri.edu.au (P.J.L.); 2 Department of Paediatrics, The University of Melbourne, Parkville 3052, Australia; 3 Institut du Cerveau-Paris Brain Institute-ICM, Sorbonne Université, Inserm, CNRS, Hôpital de la Pitié Salpêtrière, F-75013 Paris, France; sara.baldassari@icm-institute.org; 4 Murdoch Children’s Research Institute, Parkville 3052, Australia; 5 Department of Neurology, The Royal Children’s Hospital, Parkville 3052, Australia

**Keywords:** focal cortical dysplasia, hemimegalencephaly, epilepsy, mTOR signalling, genetics

## Abstract

Type II focal cortical dysplasia (FCD) is a neuropathological entity characterised by cortical dyslamination with the presence of dysmorphic neurons only (FCDIIA) or the presence of both dysmorphic neurons and balloon cells (FCDIIB). The year 2021 marks the 50th anniversary of the recognition of FCD as a cause of drug resistant epilepsy, and it is now the most common reason for epilepsy surgery. The causes of FCD remained unknown until relatively recently. The study of resected human FCD tissue using novel genomic technologies has led to remarkable advances in understanding the genetic basis of FCD. Mechanistic parallels have emerged between these non-neoplastic lesions and neoplastic disorders of cell growth and differentiation, especially through perturbations of the mammalian target of rapamycin (mTOR) signalling pathway. This narrative review presents the advances through which the aetiology of FCDII has been elucidated in chronological order, from recognition of an association between FCD and the mTOR pathway to the identification of somatic mosaicism within FCD tissue. We discuss the role of a two-hit mechanism, highlight current challenges and future directions in detecting somatic mosaicism in brain and discuss how knowledge of FCD may inform novel precision treatments of these focal epileptogenic malformations of human cortical development.

## 1. Introduction and Historical Context

Malformations of cortical development such as focal cortical dysplasia (FCD) are major causes of drug-resistant focal epilepsy, with surgical resection often being the only effective treatment. Data from the European Epilepsy Brain Bank estimated that malformations of cortical development contributed to 19.8% of specimens collected through epilepsy surgery, and 70.6% of these specimens corresponded to different FCD subtypes [1]. The relationship between cortical lesions and epileptic seizures was established by the work of Sir John Hughlings Jackson in the 19th century [2], emphasising the pathological and anatomical basis of epilepsy. This knowledge greatly influenced the way epilepsy is treated and, indeed, the first documented epilepsy surgery conducted by Sir Victor Horsley in 1886 involved the resection of a post-traumatic, epileptic cortical scar in order to control seizures [3]. As neurosurgical techniques advanced, the field of neuropathology also underwent major development since the late 19th century, including the invention of Nissl staining in 1884 [4]. However, it was not until the second half of the 20th century that FCD was recognised as a disease entity. The pioneering work published by David Taylor and colleagues in the August 1971 issue of *Journal of Neurology, Neurosurgery, and Psychiatry* represents the first formal description of FCD, marking the beginning of scientific investigation into the malformation [5]. Taylor and colleagues described histopathological findings of brain specimens from 10 individuals who underwent neurosurgery for drug-resistant focal epilepsy. The authors noted the “most striking” feature in these specimens being the “localized disruption of the normal cortical lamination by an excess of larger aberrant neurones scattered randomly through all but the first layer”. These “aberrant neurones” with “inappropriate size”, “bizarre structure”, and “pointed in all directions”, are what we know now as “dysmorphic neurons”, an abnormal cell type found in some FCD specimens. Furthermore, in seven of the 10 individuals, they reported the presence of “malformed cells of uncertain origin with large, sometimes multiple, nuclei surrounded by an excess of opalescent, pseudopodic cytoplasm”. This referred to the second abnormal cell type that can sometimes be found in FCD specimens–the “balloon cells”.

Decades after the first description, much has been learnt about the characteristics of FCD, based on which different FCD subtypes are defined. The most recent classification established by the International League Against Epilepsy [6] proposed three FCD subtypes: FCD type I (FCDI) exhibits abnormal radial and/or tangential lamination; FCD type II (FCDII) is characterised by abnormal lamination and the presence of an abnormal cell type; and when FCDI co-exists with another brain lesion, it is classified as FCD type III (FCDIII). FCDII can be further divided into FCDIIA when only dysmorphic neurons are present, or FCDIIB when there are dysmorphic neurons and balloon cells. In hindsight, the 10 individuals described by Taylor most likely had what we now call FCDII, and seven of those would be described as FCDIIB due to the presence of balloon cells.

The majority of FCD cases are sporadic, and the genetic causes underlying FCD remained unclear until relatively recently. However, with the advancement of genomic technologies and access to biological specimens, investigators are now able to explore the genetics and biology of FCD with much greater precision. This review summarises how the field reached its current understanding of FCD, with a particular focus on FCDII and its relationship with the mTOR signalling pathway. We highlight key milestones in the field in stepwise, chronological order. We begin the story with the early recognition of mTOR pathway dysregulation in FCDII, describe the discovery of somatic mosaicism in focal brain lesion and the evidence of two-hit mechanism, and finally discuss challenges in the field and future directions.

## 2. The “mTORopathy Hypothesis”—The 2000s

Early studies of FCD were, in part, informed by the knowledge gained from the analysis of tuberous sclerosis complex (TSC). TSC is a multisystem genetic syndrome characterised by benign tumours (or “hamartomas”) in multiple organs including the kidney, heart, and brain [7]. Histopathological similarities shared by TSC brain lesions known as “cortical tubers” and FCD have long been recognised since the report by Taylor [5]. In his concluding remarks, Taylor noted that while FCD should be considered as a distinct form of cortical malformation, there were undeniable similarities between the histopathological features of FCD and those of cortical tubers. In terms of histopathology, cortical tubers are comparable to FCDIIB, characterised by a disruption in cortical layering and the presence of dysmorphic neurons and balloon/giant cells. While the genetic causes of FCD remained unclear until relatively recently, early linkage studies attributed TSC to two disease-causing loci located on chromosome 9 and 16 [8,9], and the two genes were eventually identified and termed *TSC1* and *TSC2* respectively [10,11]. The protein products of *TSC1* and *TSC2*, hamartin and tuberin, form a protein complex, with early work in *Drosophila* showing that the proteins function in the mTOR signalling pathway and are tumour suppressors [12,13,14]. Given the histopathological similarities between FCD and TSC, it therefore appeared plausible that mTOR dysregulation could be involved in FCD.

**Figure 1:**
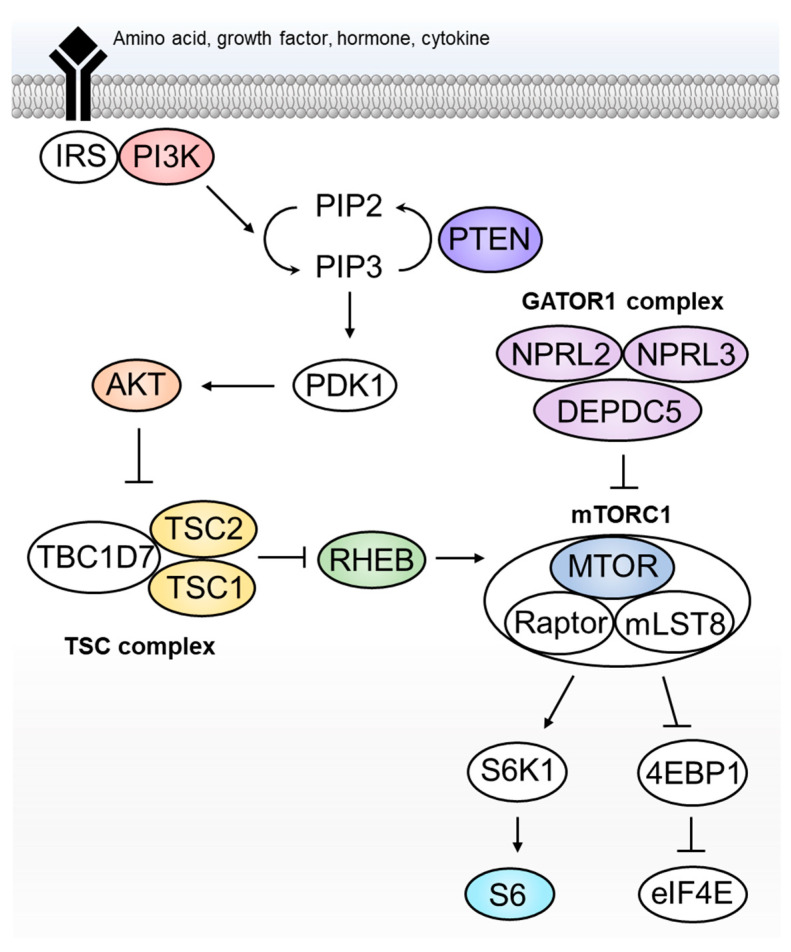
The mechanistic target of rapamycin (mTOR) signalling pathway. Pathogenic germline or somatic variants are identified in the coloured components. Specifically, *PTEN* and *S6* are associated with hemimegalencephaly (HME) only, while the rest are associated with both focal cortical dysplasia (FCD) and HME.

First purified in the 1990s by three independent groups [15,16,17], mTOR (Mechanistic Target Of Rapamycin Kinase) is a protein kinase present in the cell in two complexes, mTORC1 and mTORC2, and is a target of rapamycin, which is a compound with antifungal, immunosuppressive and anticancer properties [18]. Indeed, the identification of mTOR was mainly driven by the desire to understand the mechanism of action of rapamycin [19]. For almost three decades since the purification of mTOR, a significant amount of work has been done to elucidate the function and the mechanism of action of mTOR. We now know that the mTOR signalling pathway (whose simplified representation is reported in Figure 1) is implicated in a wide variety of cellular functions, including cellular growth, proliferation, metabolism and autophagy, by sensing and integrating various environmental and cellular cues, such as amino acids levels, growth factors, hormones and cytokines. In the brain, the mTOR pathway also has specific roles during both development and adulthood (reviewed in [20]). mTOR activity is finely controlled by several proteins acting as activators (e.g., PI3K, AKT, RHEB) or repressors of the pathway (e.g., PTEN, TSC and GATOR1 complexes) in response to the above-mentioned signals. As a result of the signalling cascade, downstream substrates and effectors such as S6K1 and 4EBP1 are phosphorylated, which in turn initiate key cellular functions such as protein and lipid synthesis through their specific molecular mechanisms (reviewed in [20]).

In 2004, *Annals of Neurology* featured two articles that utilised immunohistochemical methodologies to test the potential role of the mTOR pathway in FCD and TSC [21,22]. Both studies used the phosphorylated substrates downstream of the mTOR pathway as a proxy to assess the intrinsic status of the signalling pathway. These downstream phosphorylated substrates, including phosphorylated ribosomal protein S6 (p-S6), are a result of the serine/threonine kinase activity of mTOR [23]. The most remarkable outcome of the two studies was the consistent finding that in balloon/giant cells and dysmorphic neurons from both FCD and TSC, enhanced immunoreactivity against p-S6 was observed. This finding indicated that hyperactivation of the mTOR pathway is a common feature of TSC, and that TSC and FCD may be more closely related than originally thought.

First described in 1835 [24], hemimegalencephaly (HME) is a cortical malformation characterised by abnormal enlargement of one cerebral hemisphere, with histopathological features generally indistinguishable from those of FCDII [25,26]. Indeed, the identification of mTOR pathway dysregulation in FCD and TSC was followed shortly afterwards by corroborating findings in HME [27,28,29,30]. These studies consistently demonstrated enrichment of p-S6 immunoreactivity in HME brain specimens, suggesting that HME and FCD not only share similar histopathological features, but the two are commonly associated with the mTOR pathway. These findings further expanded the spectrum of disorders related to the mTOR pathway, a group of conditions first referred to by Crino as “mTORopathies” [31]. With the concurrent development of next generation sequencing technologies, the genetic basis of these brain malformations was further elucidated in the following decade.

## 3. Lessons Learnt from Hemispheric Malformations—2012

The sporadic occurrence of HME as a unilateral malformation suggests the involvement of postzygotic somatic mutation. This hypothesis is supported by previous report of monozygotic twins with one twin affected by HME but not the other [32]. In 2012, Lee and colleagues used whole exome sequencing (WES) on paired blood and brain specimens from five individuals with HME and identified brain-specific somatic variants in three [33]. These variants were in *AKT3* (c.49G>A; p.Glu17Lys), *PIK3CA* (c.1633G>A; p.Glu545Lys) and *MTOR* (c.4448C>T; p.Cys1483Tyr), all of which are involved in the mTOR pathway (Figure 1). Using single-base extension mass spectrometry in another 15 individuals with HME, they identified the recurrent *PIK3CA* c.1633G>A (p.Glu545Lys) variant in three. Furthermore, immunostaining showed enhanced p-S6 labelling in the brain tissue of individuals carrying variants in *AKT3*, *PIK3CA* and *MTOR*, indicating aberrant mTOR signalling pathway. Poduri and colleagues used SNP microarray and quantitative PCR to identify mosaic trisomy of chromosome 1q in two individuals with HME [34]. One of the many genes that chromosome 1q contains is *AKT3*. The involvement of *AKT3* in HME is further supported by the identification of *AKT3* c.49G>A (p.Glu17Lys) variant in an additional individual with HME [34]. These genetic findings confirmed the hypothesis that *de novo* somatic mutations are associated with focal cortical malformation, and variants in the mTOR pathway genes likely contribute to the elevated p-S6 immunoreactivity that is consistently observed in the dysplastic brain specimens.

## 4. The Curious Cases of Familial FCD, Non-Lesional Epilepsy and the Two-Hit Hypothesis—2014

Leventer and colleagues reported six families affected by FCD and related brain lesions [35], and were the first to propose that FCD may be familial in rare pedigrees. While the majority of FCD cases are sporadic, other pedigrees with multiple family members affected by FCD have since been described. These families either had multiple individuals with FCD or had one case with FCD and another family member affected by HME, ganglioglioma, or dysembryoplastic neuroepithelial tumour. Although the precise mechanism was unknown, the occurrence of FCD in a familial context suggested a germline susceptibility in these individuals. In addition, the fact that FCD and HME can occur in the same pedigree provided further support to the notion that the two disorders are closely related and may share similar genetic factors.

**Figure 2:**
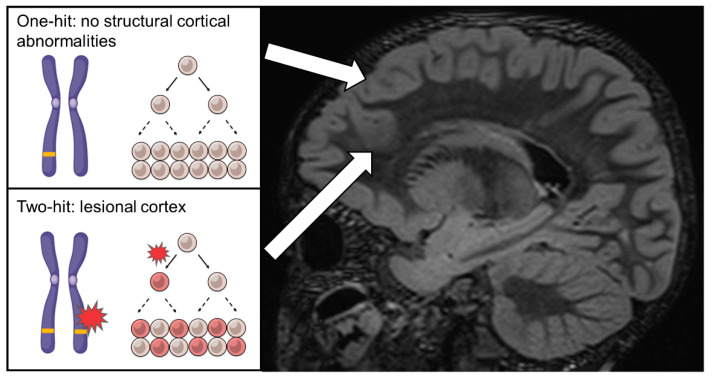
Illustration of two-hit mechanism in cortical dysplasia. In the unaffected cortex, a heterozygous germline *DEPDC5* variant is present in all cells but does not cause “visible” lesion at MRI. In the lesional cortex, it is hypothesised that a postzygotic somatic *DEPDC5* variant (or variant in another mTOR pathway genes) occurred in a subset of cells, leading to localised brain lesion.

Studies of families affected by DEPDC5-mediated epilepsy have contributed significantly to our current understanding of FCD. DEPDC5 is a component of the GATOR1 complex [36], which functions as a repressor in the amino acid-sensing branch mTOR pathway. Heterozygous germline variants in *DEPDC5* were initially implicated in non-lesional (MRI-negative) familial focal epilepsy including familial focal epilepsy with variable foci, autosomal dominant nocturnal frontal epilepsy, and familial temporal lobe epilepsy [37,38]. The majority of the identified variants were predicted to introduce premature stop codons, suggesting that haploinsufficiency of *DEPDC5* may result in different subtypes of epilepsies in the absence of a cortical malformation. However, a subsequent study showed that *DEPDC5* variants may also be associated with lesional focal epilepsy [39]. Using WES and high-resolution melting analysis, Scheffer and colleagues identified heterozygous germline *DEPDC5* variants in three unrelated families. These variants were c.418C>T (p.Gln140*), c.21C>G (p.Tyr7*) and c.279+1G>A, each found within one family. Interestingly, within the same family, carriers of the *DEPDC5* variants may show lesional or non-lesional focal epilepsy, and some may even be clinically unaffected. The reason for this incomplete penetrance was unclear. Those carriers with lesional focal epilepsy showed characteristic FCDII features on MRI, suggesting that *DEPDC5* variants may be linked to FCD. The exact mechanism was not fully understood, but the authors suggested that a two-hit mechanism, analogous to the two-hit cancer model [40] may be involved. A *DEPDC5* variant, in combination with a second hit in the other *DEPDC5* allele or another mTOR pathway gene, may lead to misregulated growth in a somatic lineage, which, in turn, results in cortical malformation (Figure 2). This two-hit mechanism has long been suggested to play a role in TSC [41,42,43], but its involvement in FCD was ascertained only recently (discussed below).

## 5. First Evidence of Two-Hit Mechanism and Somatic *MTOR* Variants in FCD—2015~2016

Between 2015 and 2016, a number of studies reported FCD associated with heterozygous germline variants in genes including *DEPDC5* [44,45,46,47,48,49], *NPRL2* [48] and *NPRL3* [48,50]. These three genes encode proteins that form the GATOR1 complex, which has an inhibitory role on the mTOR pathway [36]. The majority of these germline variants were truncating, and it was hypothesised that the loss of one functional copy of the gene (germline first hit) coupled with the loss of the second copy through a somatic second hit, could explain the occurrence of focal lesions in these individuals.

The identification of a putative somatic second hit requires access to affected brain tissue. Among the studies that reported germline variants in GATOR1 complex genes, three examined brain-derived DNA from individuals with FCD [45,46,50]. A study of two individuals with FCD and a heterozygous germline *DEPDC5* variant did not detect any somatic variants in *DEPDC5* in affected brain tissue utilising molecular inversion probe sequencing (MIPS) and WES [46]. Similarly, low coverage WES (mean depth 80X) and Sanger sequencing of brain-derived DNA from two individuals with FCD and a germline heterozygous *NPRL3* variant did not detect additional somatic variants [50]. The first evidence of a two-hit mechanism in FCD was demonstrated by Baulac and colleagues [45]. In this study, they identified heterozygous germline *DEPDC5* variants in seven individuals with FCD, two of which had FFPE brain specimens available for analysis. Using Sanger sequencing, they identified a brain-specific somatic *DEPDC5* c.1264C>T (p.Arg422*) variant in one individual in addition to the germline *DEPDC5* c.715C>T (p.Arg239*) variant detected in blood and brain [45]. This finding demonstrated a two-hit mechanism in FCD, although the allelic configuration of the germline and somatic variants and therefore the proof of a bi-allelic inactivation could not be ascertained due to suboptimal quality of FFPE tissue-derived DNA [45]. In another study, a somatic *DEPDC5* frameshift variant (allele frequency 35% in brain, 28% in saliva) and a brain-specific, mosaic copy-neutral loss of heterozygosity affecting chromosome 22 was identified in an individual with HME [51]. This suggested that functional inactivation of both *DEPDC5* copies in the brain could be involved. However, it was unknown whether the frameshift mutation and the loss of heterozygosity acted in *trans*, and indeed whether the loss of heterozygosity on chromosome 22 involved the genomic location of *DEPDC5* at all. Although a definite conclusion of a bi-allelic two-hit mechanism could not be established, these findings provided valuable insights into the possible involvement of bi-allelic two-hit in cases identified with germline variants in GATOR1 complex genes.

In part driven by the discoveries in HME, the involvement of *MTOR* variants in FCD was simultaneously explored by multiple centres with access to surgical brain tissue. Between 2015 and 2016, multiple teams identified low allele frequency somatic *MTOR* variants in brain-derived gDNA from individuals with FCD using WES and/or targeted sequencing [51,52,53,54,55]. A number of recurrent cancer hotspot *MTOR* variants were commonly found in these studies, including c.6644C>T (p.Ser2215Phe), c.6644C>A (p.Ser2215Tyr) and c.4379T>C (p.Leu1460Pro), all of which were previously found to confer mTOR pathway hyperactivation [56]. Collectively, these studies confirmed the hypothesis that FCD can be caused by somatic variants in genes involved in the mTOR pathway. Given that variant allele frequencies as low as 1% were identified, the application of deep sequencing was an essential component of the experimental design. All the identified *MTOR* variants were missense, predicted to result in constitutive activation of the protein and, therefore, hyperactivation of the signalling pathway. Increased levels of p-S6 were detected by immunohistochemistry and Western blot analyses of brain-derived tissue or analysis of cultured cells overexpressing mutant proteins. To explore the effects of mosaic *MTOR* somatic variant on brain development, one study utilised in utero electroporation (IUE) to introduce the mutant *MTOR* transcript focally in the cortex of E14 mouse embryos, thereby recapitulating the focal nature of FCD lesion [52]. Spontaneous seizures, disrupted neuronal migration and the occurrence of cytomegalic neurons (that resemble dysmorphic neurons) were observed in these mice. Furthermore, the spontaneous seizures and cytomegalic neurons could be rescued by intraperitoneal administration of rapamycin, a drug capable of repressing the mTOR pathway. Collectively, these findings suggested a crucial role for mTOR pathway hyperactivation in FCD secondary to brain-specific somatic *MTOR* variants.

In addition to *MTOR*, somatic variants in two other mTOR pathway genes, *PIK3CA* and *AKT3*, were implicated in FCD [57]. In an individual affected by FCD, a *PIK3CA* c.3140A>G (p.His1047Arg) variant was identified as a brain-specific somatic variant with an allele frequency of 4.7%. Of note, the same variant was identified in gDNA derived from both brain and peripheral blood of an individual with HME with an allele frequency of 13% [46], suggesting that the same variant could result in either FCD or HME. Collectively, these findings further support the notion that FCD and HME share very similar genetic etiologies and studying one disorder may therefore help delineate the biology and pathomechanisms of the other.

## 6. Somatic Mosaicism in *TSC1/2* and the Continuum of Cortical Dysplasias—2017

The involvement of the mTOR pathway in FCD was further supported by the identification of somatic variants in *TSC1* and *TSC2*. Analysis of 40 individuals with FCD (previously screened and found to be negative for variants in *MTOR* gene) [52] identified two somatic *TSC1* variants [c.610C>T (p.Arg204Cys) and c.64C>T (p.Arg22Trp)] in four individuals and one somatic *TSC2* c.4639G>A (p.Val1547Ile) variant in one individual using targeted hybrid capture and PCR-based amplicon sequencing [58]. These missense variants were brain-specific, and the variant allele frequencies were less than 3% in all cases. Study of cultured cell lines transiently expressing the mutant proteins showed that these missense variants affected the formation or function of the TSC1-TSC2 complex, thereby inducing hyperactivation of the mTOR pathway. The authors also performed two IUE experiments in mice to investigate the in vivo effects of focal disruption of TSC1 or TSC2. The first IUE experiment used Cas9 nuclease to introduce indels in *Tsc1* or *Tsc2*, thereby generating mice with focal knockout of either gene. IUE was performed at E14 and at >21 days of age half of the mice exhibited spontaneous seizures, defective neuronal migration, and cytomegalic neurons, similar to mice focally expressing mutant *MTOR* [52,58]. The second IUE experiment aimed to investigate if focal expression of a disease-associated human *TSC2* c.4639G>A (p.Val1547Ile) variant could lead to FCD phenotypes in mice. This was achieved by exploiting Cas9 nickase-mediated homology directed repair to focally introduce the corresponding mouse *Tsc2* c.4576G>A (p.Val1526Ile) variant in the brain. IUE was performed at E14 and examination of mice at 28 days of age showed cytomegalic neurons in the brain, and seizures in 50% of the mice. Taken together, these IUE experiments suggested that focal brain knockout in *Tsc1* or *Tsc2* genes in mice can cause phenotypes similar to FCD, and mosaic substitution of disease-associated missense variant can indeed result in the formation of cytomegalic neurons in vivo.

The finding that somatic *TSC1* and *TSC2* variants could cause FCD was rapidly corroborated by a subsequent study in which two individuals with FCD were reported with a somatic *TSC1* c.163C>T (p.Gln55*) variant and a somatic *TSC2* c.2251C>T (p.Arg751*) variant [59]. These variants were predicted to introduce premature stop codons, thus confirming that a loss of function mechanism underlies TSC and FCD linked to TSC1 and TSC2 variants, suggesting that in addition to missense variants described previously [58]. Of note, this study also reported two individuals with HME carrying *TSC2* variants. The first individual had a heterozygous germline *TSC2* c.1892T>C (p.Leu631Pro) variant and a somatic *TSC2* c.4672G>A (p.Glu1558Lys) variant; while the second individual had a heterozygous germline *TSC2* c.5138G>A (p.Pro1713His) variant and a somatic *TSC2* c.1754_1755delGT (p.Tyr587*) variant [59]. This raised the possibility that a two-hit mechanism may be involved in HME, although the allelic configuration was not reported. Despite the finding of germline and somatic variants in *TSC2*, the typical clinical features of TSC were not observed in the two individuals with HME. Interestingly, a subsequent study identified a heterozygous germline *TSC1* c.90delA (p.Glu31Argfs*12) variant in two related individuals affected by FCDIIB without any features of TSC [60]. The pathomechanism was unclear and the presence of somatic *TSC2* variants in the two individuals was not able to be confirmed, as these individuals did not undergo neurosurgery. Together, these findings suggest that genetic variants in *TSC1* or *TSC2* may not necessarily lead to TSC but can result in isolated FCD and HME when they occur during neurodevelopment and affect only a subset of brain cells.

**Figure 3:**
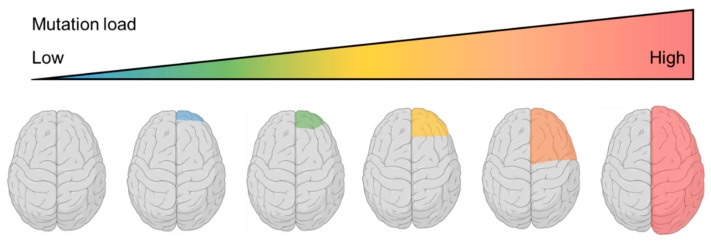
Focal cortical dysplasia (FCD) and hemimegalencephaly (HME) as a continuum of cortical dysplasias caused by commonly shared genetic variants with different mutation load. In small FCD, a small subset of cells is affected, which contribute to the small mutation load and restricted lesion. In HME, a larger number of cells carry the somatic variant, resulting in higher mutation load and a more diffuse lesion. It is important to acknowledge that there are even smaller lesions including presumed MRI-negative lesions within this spectrum, associated with ultra-low mutation load.

FCD and HME are primarily distinguished by their neuroimaging features, with FCD typically involving a relatively smaller cortical area and HME affecting one cerebral hemisphere more diffusely. It has been proposed that the extent of brain malformation is determined by the timing of the genetic insult [61]. A somatic mutation arising earlier during the development may result in a larger proportion of mutated cells and hence a more severe brain lesion such as HME, while a somatic mutation occurring at later proliferative stages may lead to a more subtle brain lesion such as FCD. The concept of a “continuum of cortical dysplasias” was proposed (Figure 3), using a group of individuals with brain malformations as an example [59]. These individuals exhibited different extents of lesions, ranging from FCD to HME to polymicrogyria with macrocephaly. By comparing the genetic and phenotypic findings, the authors illustrated that FCD typically has lower somatic mutation load when compared to HME, while polymicrogyria with macrocephaly, which has a more diffuse phenotype, is associated with heterozygous germline mutation. There is also a marked overlap between the level of mutation load between FCD and HME, suggesting that these conditions represent a “continuum of cortical dysplasias” secondary to distinct timing of genetic mutations during development.

## 7. Confirmation of the Two-Hit Mechanism in FCD and the Identification of Somatic *RHEB* Variants—2018~2019

The long-standing hypothesis of the two-hit mechanism in FCD was proven in 2018 by Baulac and colleagues. Deep sequencing of paired blood and brain-derived gDNA samples from 10 individuals with FCD identified one case with a heterozygous germline *DEPDC5* c.856C>T (p.Arg286*) variant and a somatic *DEPDC5* c.865C>T (p.Gln289*) variant [62]. The heterozygous germline variant was inherited from an unaffected mother, whereas the brain-specific somatic variant was only present in the brain tissue. A mutation gradient with a higher rate of mosaicism in the seizure-onset zone (~10%) than in the surrounding epileptogenic zone (~0.3%) was observed. Examination of sequencing reads showed a *trans* configuration between the two variants, indicating that bi-allelic inactivation of *DEPDC5* in a subset of cells was involved. Focal knockout of *Depdc5* in mice using IUE and CRISPR-Cas9 resulted in spontaneous seizures, impaired neuronal migration, cytomegalic p-S6-positive neurons, and altered neuronal electrophysiology, highlighting the causal link between the focal loss of DEPDC5 function and the FCD phenotype. Notably, 30% of these mice exhibited spontaneous seizure followed by sudden death, a phenomenon closely resembling sudden unexpected death in epilepsy (SUDEP), reported in some individuals with *DEPDC5*-related epilepsy [48,63,64,65]. Administration of rapamycin into pregnant dams prevented the neuronal migration defect in mouse embryos with focal *Depdc5* knockout. Taken together, this study provided proof-of-concept that the two-hit model could contribute to FCD in individuals with a heterozygous germline variant.

Following the initial confirmation of a two-hit event in FCD, additional cases have been identified with the same mechanism [66,67,68]. A germline *DEPDC5* c.2390delA (p.Gln797Argfs*18) variant and a somatic *DEPDC5* c.3994C>T (p.Arg1332*) variant were identified in an individual with FCDIIA, with laser capture microdissection demonstrating the enrichment of the somatic variant in dysmorphic neurons [66]. In an individual with hemispheric FCDIIA, a heterozygous germline *DEPDC5* c.3021+1G>A variant was identified by targeted sequencing. Further investigation of resected brain tissue suggested a loss of heterozygosity of this *DEPDC5* allele in the brain [67]. Sanger sequencing analysis of gDNA derived from laser capture microdissected brain cells demonstrated that normal neurons and glial cells were heterozygous for the variant, whereas dysmorphic neurons showed high enrichment of the mutant allele, with the wildtype allele almost undetectable [67]. Another study identified a germline *DEPDC5* c.4521_4522del (p.Thr1508Serfs*58) variant and a somatic *DEPDC5* c.4189_4196dup (p.Phe1399Leufs*21) variant in an individual with FCDIIB, suggesting that the two-hit mechanism is not specific to FCDIIA [68]. However, it is notable that most reported cases with *DEPDC5* variant were associated with FCDIIA. Collectively, these studies highlight the involvement of a two-hit mechanism in FCD, although they currently represent only a small proportion of genetically diagnosed cases. Furthermore, genetic events leading to loss of heterozygosity are not readily detectable using next-generation sequencing techniques, suggesting that a potentially significant proportion of cases with a two-hit mechanism remain to be identified.

The two-hit mechanism may also involve two distinct genes, instead of having two affected alleles in one gene. For example, a somatic *MTOR* c.6644C>T (p.Ser2215Phe) variant found only in brain and a mosaic *RPS6* c.695G>A (p.Arg232His) detected in brain and blood, were identified in an individual with HME [69]. A single somatic *MTOR* c.6644C>T (p.Ser2215Phe) variant was previously reported as sufficient to cause both HME and FCD [51,52,54,55,59]. To investigate the effects of the double variants, the authors performed IUE on rat embryos at E15 to overexpress the mutant MTOR p.Ser2215Phe protein or the mutant RPS6 p.Arg232His protein or both mutant proteins. Rats producing both mutant proteins exhibited more severe phenotypes compared to rats producing single mutant protein, with increased proliferation and migration defects at the embryonic stage and occurrence of cytomegalic cells at the postnatal stage. The authors proposed that the presence of both variants had synergistic effects on cortical development. Notably, another study reported an individual with TSC carrying a heterozygous germline *TSC2* c.2355+2T>A variant and a somatic *DEPDC5* c.2845C>T (p. Arg949Cys) variant [68]. However, the specific effects of the two variants were not functionally characterised. The “two genes two-hit” mechanism in the context of FCD warrants further investigation. In particular, it is critical to assess whether both variants were present in the same cells.

Somatic *RHEB* variants have recently been implicated in FCD and HME. A somatic *RHEB* c.119A>T (p.Glu40Val) variant was initially reported in an individual with HME [70]. Immunohistochemistry demonstrated p-S6 immunoreactivity in brain sections, suggesting upregulation of the mTOR pathway. Similarly, a somatic *RHEB* c.104_105delACinsTA (p.Tyr35Leu) variant was identified in an individual with FCDIIA, and aberrant activation of the mTOR pathway was confirmed by Western blot and immunohistochemistry [71]. The authors also performed IUE on E14.5 mouse embryos to introduce the mutant RHEB p.Tyr35Leu protein focally in the cortex. Examination of E18.5 mouse embryos and P30 mice producing the mutant RHEB protein showed altered neuronal migration and cytomegalic neurons with p-S6 immunoreactivity in the cortex. Importantly, spontaneous tonic-clonic seizures were observed in P30 mice, and this was alleviated by administration of rapamycin [71]. Another study also reported a somatic *RHEB* variant in a case with HME [c.104_105delACinsTA (p.Tyr35Leu)] and another in a case with FCDIIB [c.119A>T (p.Glu40Val)], providing further support to the pathogenic role of *RHEB* variants [67]. Taken together, these findings identified *RHEB* as an additional “mTORopathy” causal gene for FCD and HME. In light of this novel observation, it is possible that variants in other, yet to be identified mTOR pathway genes, also contribute to FCD.

Two large cohort studies explored the genetics of FCD in a systematic manner. The first study by Baulac and colleagues analysed matched brain-blood tissues of 62 individuals with FCDII or HME [67] and identified pathogenic variants in ~60%: somatic *MTOR* variants in 20 (32.3%), somatic *PIK3CA* variants in four (6.5%), somatic *AKT3* variant in three (4.8%), somatic *TSC1* variants in two (3.2%), somatic *TSC2* variants in two (3.2%), germline *TSC2* variant in one (1.6%), somatic *RHEB* variants in two (3.2%), germline *DEPDC5* variants in three (4.8%), and two-hit *DEPDC5* variants in two (3.2%). The authors further showed that panel-negative FCDII cases also display p-S6-positive dysmorphic neurons and balloon cells, suggesting the existence of a hitherto undetected somatic variant in an mTOR pathway gene. By demonstrating the presence of somatic variants specifically in dysmorphic neurons and balloon cells, this study emphasized the link between neuropathology and genetics. The second study led by Lee and colleagues investigated brain tissue of 114 individuals with FCDII or HME [68], and identified pathogenic somatic *PIK3CA* variant in one (0.9%), somatic *AKT3* variant in one (0.9%), somatic *TSC1* variants in six (5.3%), somatic *TSC2* variants in two (1.8%), somatic *MTOR* variants in twenty-four (21.1%), germline *DEPDC5* variant in four (3.5%), and two-hit *DEPDC5* variants in one (0.9%). These studies provided insights into the genetic landscape of cortical dysplasia, highlighting the significance of mTOR pathway in FCDII and HME.

## 8. Pathomechanism of FCDII: What Have We Learnt to Date?

The mTOR pathway was first recognised to be involved in FCD in early 2000s, but the precise genetic factors underlying FCD have only started to be unravelled in recent years. Despite the slow start, the development of the field was rapid. Between 2014–2019, the field has discovered that both single-hit somatic mosaicism and two-hit germline plus somatic mutations can contribute to the pathogenesis of FCD. These mTOR pathway genes, including *AKT3*, *PIK3CA*, *MTOR*, *RHEB*, *TSC1*, *TSC2*, *DEPDC5*, *NPRL2* and *NPRL3* explain up to 60% of FCDII cases to date. Morphologically similar brain malformations, such as HME and cortical tubers of TSC, are also associated with dysregulation of the mTOR pathway, highlighting the importance of this pathway in brain malformations. It is important to acknowledge that *SLC35A2*, a gene involved in N-glycosylation, has recently been associated with FCDI and mild malformation of cortical development with oligodendroglial hyperplasia in epilepsy (MOGHE) [67,68,72,73,74,75]. These cases are distinct from FCDII in terms of histopathology and the perturbed molecular pathway, and hence are not discussed in this review.

Despite the progress in understanding the molecular genetics of FCD, its pathomechanism and the means by which FCD is epileptogenic remain incompletely understood. It has been known for some time that the dysmorphic neurons are the “seizure generating” cells in type II FCD and TSC [76] based on electrophysiological studies of resected FCD tissue prior to knowledge of FCD genetics. With somatic mosaicism being a significant contributor to cortical dysplasia, several studies have investigated the cell types harbouring the somatic variants, and the effects of somatic mutation load on electrophysiological phenotype. Single-cell DNA sequencing of brain specimens derived from individuals with HME or FCD showed that the pathogenic somatic variants can occur in both neuronal and non-neuronal lineage, but in small FCD cases they occur specifically in the neuronal lineage only [59]. Using laser capture microdissection, other studies have shown that somatic variants in HME and FCD were most highly enriched in dysmorphic neurons and/or balloon cells compared to other cell types [66,67,77]. Furthermore, the density of dysmorphic neurons and balloons cells in the FCD brain specimens was positively correlated with the level of somatic mutation load [66,67]. In an individual with FCDIIA, somatic mutation load in five brain regions were analysed, with the highest mutation load identified in the region with frequent, high voltage spike-waves and fast ripples [66]. Interestingly, IUE mouse models producing a constitutively active form of Rheb (Rheb^CA^) at low, intermediate, and high levels displayed mTOR hyperactivation, increased neuronal soma size, and neuronal misplacement in a dosage dependent manner [78]. While low Rheb^CA^ level did not induce seizures, intermediate and high Rheb^CA^ levels resulted in spontaneous and recurrent seizures increasing proportionally with higher dosage [78]. This finding suggested that different levels of mTOR hyperactivity can alter the electrophysiological phenotypes in a mouse model. In individuals affected by FCD, regional differences in somatic mutation load presumably lead to varied levels of mTOR dysregulation, which may explain the electrophysiological findings.

So far, the identified genes are predominantly involved in the mTOR pathway, but the pathomechanism may be broader than a primary mTORopathy. For example, using in vitro cultured cell and in vivo IUE mouse models, Park and colleagues [79] found that mutant MTOR p.Cys1483Tyr protein caused inhibition of cellular autophagy and aberrant accumulation of autophagy substrate OFD1, thereby disrupting the development of primary cilia, a key organelle for the integration of extracellular signalling pathways [80,81,82,83]. The authors further showed that disrupted neuronal ciliogenesis led to cortical dyslamination in IUE mutant mice by abrogating Wnt signalling, which is responsible for neuronal polarization and proper cortical lamination [84]. Notably, rescue of ciliogenesis by knocking down *Ofd1* in IUE mutant mice restored Wnt signalling and cortical lamination, but did not reverse spontaneous seizures or cytomegalic neurons, suggesting that cortical dyslamination is not necessary for epileptogenesis in IUE mutant mice [79]. Another study by Hsieh and colleagues confirmed that seizures are not due to cortical dyslamination. The authors generated an IUE mouse model producing Rheb^CA^, which successfully recapitulated FCD phenotypes including spontaneous seizures, cortical dyslamination and cytomegalic neurons [85]. Using an inducible Cre-LoxP system, production of Rheb^CA^ in mice was activated at 7 days of age when neuronal migration is mostly completed. Examination at 3–4 months showed that while cortical lamination was not affected by late production of Rheb^CA^, the mice exhibited spontaneous seizures and cytomegalic neurons, supporting the notion that neuronal misplacement may not be required for seizure generation [85]. A recent study added further insights into the mechanism of epileptogenesis in FCD by demonstrating that neurons carrying somatic *MTOR* variants can cause hyperexcitability in nearby non-mutated neurons in a non-cell autonomous manner [86].

Analyses of microRNA expression in individuals with FCD have provided some insights into the potential pathogenic alteration in FCD. microRNAs analysis by microarray found three significantly downregulated microRNAs (let-7f, miR-31, and miR-34a) in FCD brain tissue compared to control [87]. By analysing the predicted target of these microRNAs, the authors found abnormal upregulation of *NEUROG2*, which encodes a transcription factor involved in neuronal differentiation, suggesting that a disrupted differentiation process in neurons may underlie FCD [87]. Similarly, two other studies used microRNA microarray analysis to identify differentially expressed microRNA in FCD brain tissue, and suggested that some microRNAs may influence not only mTOR signalling pathway, but also LIS1 [88] and Hippo pathways [89]. In addition to analysis of microRNA in brain tissue, there have also been studies on microRNA derived from blood serum, due to its potential usage as a biomarker for epilepsy [90]. Identification of microRNA candidates in FCD brain tissue followed by validation of the candidates in serum-derived microRNA found miR-4521 [91] and miR-323a-5p [92] to be upregulated in both FCD brain tissue and serum, suggesting their potential as biomarkers for FCD.

Identification of novel genes or pathways involved in FCD may help us better understand the biology of FCD. While the involvement of environmental factors such as viral infection [93,94] cannot be excluded, these factors alone are insufficient to explain the remaining cases without a genetic diagnosis. The role of non-coding variants should be considered, and interrogation of these variants will require deep WGS to examine non-coding regions. Notably, in brain tissues from individuals affected by FCDII or HME without a genetic diagnosis, p-S6 immunoreactivity can still be observed [67], suggesting that other genes directly or indirectly related to the mTOR pathway may be involved. To delineate the genetic causes of the remaining negative cases, investigators will likely need more advanced and innovative methods. The next section highlights the current challenges in identifying somatic variants, and how we may overcome these issues with novel strategies.

## 9. Detection of Somatic Mutation: Challenges and Future Directions

**Figure 4:**
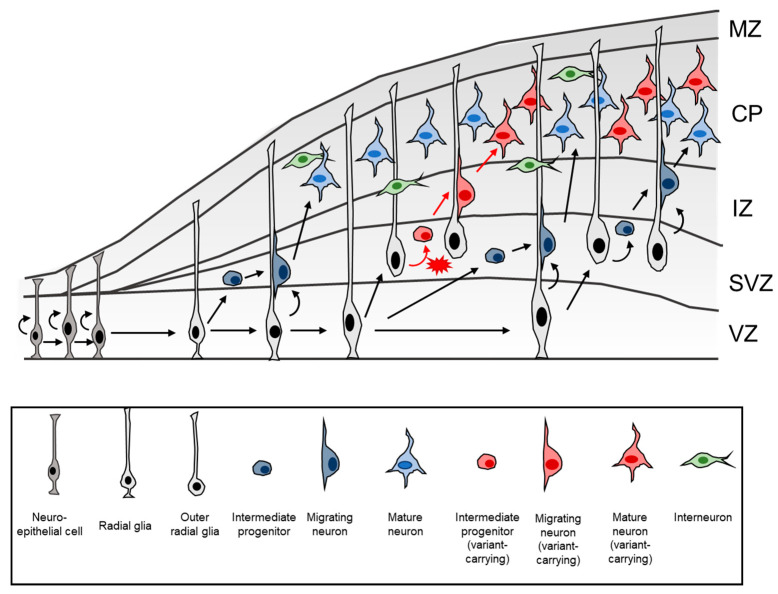
Cortical development and hypothetical occurrence of somatic variant in a subset of brain cells during neurogenesis. During cortical development, neuroepithelial cells undergo symmetric division to generate more progenitors. Neuroepithelial cells then convert into radial glia, which are able to divide asymmetrically to generate intermediate progenitor cells, migrating neurons, and more radial glia. Intermediate progenitor cells can divide symmetrically to generate clones of neurons. Neurons migrate radially along radial glia to the designated cortical layer to become mature neurons. Radial glia also give rise to outer radial glia, which also have the potential to undergo asymmetric division. In a hypothetical scenario, a somatic variant may occur in intermediate progenitor cells during the process of proliferation (red). These variant-carrying intermediate progenitor cells give rise to neurons that similarly harbour the variant. Neurons of a different clonal origin can continue to be produced normally without somatic variant (blue). This process results in a mixture of brain cells in which only a proportion of cells may carry the somatic variant. Furthermore, interneurons migrate tangentially from the ganglionic eminence (not shown), astrocytes, oligodendrocytes and microglial cells further complicate the clonal diversity in any given cortical region.

The identification of somatic variants is currently thought to be limited by low variant allele frequency, which may be below the limit of detection of current sequencing technologies. The occurrence of these rare somatic variants seen in FCD (typically < 5%) is, in part, due to the nature of cortical development [95]. During corticogenesis, pyramidal neurons migrate long distances in a radial fashion, from the ventricular zone to the different layers of cortex [96]. In contrast, interneurons originate from ganglionic eminence and migrate in a tangential direction before entering the cortex [97]. The nature of this migration process results in an interspersed population of cells from different origins [98]. Although a clone of cells harbouring the somatic mutation will be somewhat clustered, these cells intermingle with wildtype cells of diverse clonal origins, making the variant allele frequency within any given dysplastic brain sample relatively low. Furthermore, unlike proliferative cancerous tumours, whereby cells harbouring the pathogenic mutation over-represent themselves through proliferation, FCD lesions do not usually have enhanced proliferative potential. As a result, in the context of FCD, cells carrying somatic mutations are “embedded” within a network of normal cells, making the detection of a somatic mutation challenging (Figure 4).

Successful detection of rare somatic variants in brain tissue relies on the selection of appropriate brain specimens, both in terms of pathology and DNA preservation. In studies where multiple tissue samples were collected from the same individual, the concept of a “mutation gradient” was discussed [51,62,66,74]. The term “mutation gradient” refers to the regional difference in somatic mutation load between specimens from alternative cortical locations. Such a gradient can have a relatively small range, with somatic *MTOR* variants ranging from 1.2–8.6% and 1.8–7.9% mutant load in two individuals with FCD [51]. Similarly, somatic *DEPDC5* variants ranged from 0.3–10% and 0.2–3.9% mutant load in two individuals with FCD [62,66]. Indeed, this finding is consistent with the microscopic observation that abnormal cells of FCD can appear as clusters on brain sections [99,100], suggesting that cells harbouring the somatic mutation can congregate at certain regions. The regional difference in mutation load can impact the probability of successfully identifying a low frequency variant. It has been observed that brain tissue derived from the epileptogenic zone, which is defined as the minimum area of tissue needed to be resected for seizure control [101], is associated with higher somatic mutation load [51,62,66,74]. The epileptogenic zone presumably contains a higher density of dysmorphic neurons, as these cells were suggested to be the major cell type contributing to epileptogenicity [76]. Capturing the region with maximal epileptic discharges and the highest density of dysmorphic neurons can, therefore, increase the chance of detecting rare somatic mutations. This can be made possible through retrospective examination of electrophysiological data and histopathological findings when selecting brain specimens for sequencing analysis. A recent study in TSC provided proof of principle data showing that the density of dysmorphic neurons is highest in the seizure generation site [102].

To address the limitation of low variant allele frequency, one can potentially isolate a pool of abnormal cells and thereby increase the probability of detection. Using laser capture microdissection, an enriched pool of dysmorphic neurons can be collected for genetic analysis. Indeed, this technique has been successfully applied to target specific cell types from cases with a known somatic variant for downstream analyses [66,67,77]. For detection of an unknown somatic variant (variant discovery) in laser-captured cells, a WES or targeted sequencing approach will likely be required. Although the amount of gDNA extracted from a small number of laser captured cells may not be sufficient for next generation sequencing, this may be overcome by the application of whole genome amplification technology [103], which is able to amplify very low amounts of DNA to levels appropriate for downstream analyses. Indeed, previous studies have demonstrated the utility of single neuron WGS enabled by whole genome amplification methods [104,105].

While laser capture microdissection is useful in isolating specific cell types and allows morphology correlates, it is a low-throughput and laborious method may not be suitable for large scale assays. To this end, fluorescence–activated cell sorting (FACS) aided by marker-specific antibody may represent a feasible option to isolate cells of interest expressing a specific marker at a much higher throughput. In addition to the well-known p-S6 protein, recently identified markers including HCN4 [106] and FLNA [107] may also be used to select for the abnormal cell types in FCD. This approach requires, however, the use of fresh tissues (and is therefore difficult to apply in a diagnostic setting) or formalin-fixed paraffin embedded (FFPE) tissues (which lead to a poorer DNA quality, possibly reducing the chances of confidently identify pathogenic variants) [108]. A similar technique, fluorescence-activated nuclei sorting (FANS) in which nuclei are isolated instead of cells, thus allowing the use of frozen tissues, may be used to investigate somatic mosaicism in brain malformation with the aid of anti-NeuN antibody, for example [59,109]. From a variant-discovery standpoint, one could potentially isolate a large number of labelled cells/nuclei using FACS/FANS and perform sequencing analysis to screen for somatic variants in novel genes.

## 10. The Hope of Precision Treatments

Efforts to understand FCD are driven by the desire to discover targeted therapeutic interventions that could eventually improve patients’ quality of life. Converging lines of evidence have shown that mTOR hyperactivation plays a significant role in the pathogenesis of FCDII, highlighting the therapeutic potential of mTOR inhibition in patients. Indeed, treatment with the mTOR inhibitor, rapamycin, has been shown to alleviate a number of FCD-like phenotypes in IUE mouse models, including seizures [52,58,62,71,85]. EXIST-3, a clinical trial of mTOR inhibitor everolimus in 366 participants with TSC, showed some efficacy in reducing seizure frequency, with 40% response rate in individuals treated with high-exposure everolimus, 28.2% response rate in individuals with low-exposure everolimus, and 15.1% response rate in the placebo group [110]. Data of everolimus treatment in individuals with FCD is lacking, but clinical trials based in New York (NCT02451696) and Seoul (NCT03198949) are currently at Phase 2. Notably, while rapamycin treatment in an individual with HME was able to reduce seizure frequency by 50% [111], another individual with HME did not respond to everolimus treatment [112], although mosaic *MTOR* variants were found in both cases. It must be remembered that mTOR inhibitors cannot remove the presence of dysmorphic neurons, so any therapeutic potential relies on an effect of epileptogenicity. Only surgery or emerging destructive therapies such as laser ablation can remove or destroy the seizure generating dysmorphic neurons. The therapeutic potential of mTOR inhibitor warrants further investigation.

In addition to targeting the MTOR protein using rapalogs (rapamycin and its derivatives), compounds that act downstream of MTOR, or other targets associated with FCD may also have beneficial effects. Translational profiling using Ribo-Seq led to the identification of eIF4E and ADK as potential new therapeutic targets [113]. Pharmacological inhibition of eIF4E using metformin, and ADK using 5-ITU resulted in alleviation of seizures in IUE mouse model producing mutant MTOR protein [113]. Another study reported aberrant level of FLNA in FCDII brain tissue, and pharmacological inhibition of FLNA using PTI-125 was able to reduce seizure frequency in IUE mouse model producing RHEB^CA^ [107]. It is noteworthy that the ectopic expression of HCN4, a hyperpolarization-activated cyclic nucleotide-gated potassium channel, was recently associated with epileptogenesis in FCDII [106]. Although a HCN4-specific compound was not available, blocking HCN4 activity through the expression of nonfunctional HCN4 subunits successfully prevented seizure generation in IUE mouse model producing RHEB^CA^ [106]. Collectively, these studies raise the possibility of utilising compounds other than rapalogs to achieve similar or perhaps better efficacy in treating seizures associated with mTORopathy.

The presurgical identification of somatic variants in FCD and HME would further contribute to a better diagnostic and therapeutic workup for patients with drug-resistant epilepsy. Two recent studies [114,115] provided the first evidence that brain-specific somatic variants could be detected in cell-free DNA from cerebrospinal fluid samples from patients with refractory epilepsy, thus opening the way towards novel presurgical genetic testing.

## 11. Concluding Remarks

Fifty years after the initial description of FCD [5], the field is now able to dissect the biology of FCD at unprecedented resolution using modern molecular techniques. In particular, the past decade has seen remarkable advances in our understanding of the molecular genetics of FCD, enabled by deep targeted sequencing and whole exome sequencing, and application of genetic and genomic techniques directly to pathological tissue, as has been the model of investigation for cancer genetics for some decades. However, there remains a significant proportion of cases with unknown genetic cause(s), and ultra-rare pathogenic mosaic variants remain a challenge to overcome. Advancement of genomic technologies, such as single-cell sequencing and long read sequencing, may help provide novel insights into the genetics of FCD in the future. In addition to “what” causes FCD, the field may gain further insights into the precise cellular mechanism of “how” FCD occurs and results in seizures. Better understanding of the molecular mechanisms underlying FCD is needed in order to deliver improvements in both medical and surgical treatments.
